# Smad3-dependent CCN2 mediates fibronectin expression in human skin dermal fibroblasts

**DOI:** 10.1371/journal.pone.0173191

**Published:** 2017-03-07

**Authors:** Trupta Purohit, Zhaoping Qin, Chunji Quan, Zhenhua Lin, Taihao Quan

**Affiliations:** 1 Department of Pathology, University of Michigan Medical School, Ann Arbor, Michigan, United States of America; 2 Department of Dermatology, University of Michigan Medical School, Ann Arbor, Michigan, United States of America; 3 Department of Pathology & Cancer Research Center, Yanbian University Medical College, Yanji, China; University of Bergen, NORWAY

## Abstract

The potential involvement of connective tissue growth factor (CCN2/CTGF) in extracellular matrix (ECM) production is recognized. However, the role CCN2 in fibronectin (FN) gene expression has remained incompletely understood and even controversial. Here we report that CCN2 is absolutely necessary for FN expression in primary human skin dermal fibroblasts, the major cells responsible for ECM production in skin. Gain- and loss-of-function approaches demonstrate that CCN2 is an essential component of FN expression in both basal and stimulation by TGF-β signaling, the major regulator of FN expression. CCN2 is significantly induced by Smad3, a critical mediator of TGF-β signaling. CCN2 acts as a downstream mediator of TGF-β/Smad signaling and acting synergistically with TGF-β to regulate FN gene expression. Finally, we observed that CCN2 and FN predominantly expressed in the dermis of normal human skin, stromal tissues of skin squamous cell carcinoma (SCC), and simultaneously induced in wounded human skin *in vivo*. These findings provide evidence that CCN2 is responsible for mediating the stimulatory effects of TGF-β/Smad on FN gene expression, and attenuation of CCN2 expression may benefit to reduce fibrotic ECM microenvironment in disease skin.

## Introduction

Connective tissue growth factor (CTGF) is a second member of the CCN protein family (CCN2), which has six members designed CCN1 to CCN6 [1]. The CCN acronym is taken from the names of the first three members of the family to be discovered: Cyr61/CCN1 (cysteine-rich protein 61), CTGF/CCN2 (connective tissue growth factor) and NOV/CCN3 (nephroblastoma overexpressed gene). CCN2 is cysteine-rich, secreted matricellular protein. CCN2 exhibits diverse biological activities *in vitro*, such as cell proliferation, adhesion, migration, and extracellular matrix (ECM) production [2–4]. CCN2 is primarily regulated by transforming growth factor-β (TGF-β) in human skin fibroblasts [5, 6]. It appears that CCN2 functions as downstream mediator of TGF-β signaling to stimulate collagen synthesis. However, CCN2 does not directly regulate collagen synthesis. Several lines of evidence indicate that CCN2 is markedly elevated in numerous fibrotic disorders, involving skin, lungs, and kidneys, where it is believed to stimulate excessive deposition of collagen [7–10]. CCN2-null mice die shortly after birth primarily due to respiratory failure caused by skeletal defects, indicating that CCN2 plays a crucial role in regulation of cartilage ECM remodeling during development [11].

Although the potential involvement of CCN2 in collagen synthesis has been recognized [6, 12, 13], its precise biological function in other ECM synthesis has remained incompletely understood and even controversial. For instance, TGF-β-induced fibronectin (FN) expression was effectively blocked with either specific CCN2 antisense oligodeoxynucleotides [14] or CCN2 knockout cells [15]. On the other hand, mice were infected with adenoviruses encoding wild-type murine CCN2 induced strong fibrotic response in skeletal muscle by increasing ECM production including FN [16]. However, Mori et al recently reported that CCN2-null mouse embryonic fibroblasts retain intact TGF-β responsiveness including stimulation of FN and collagen by TGF-β [17].

We report here that CCN2 is a critical endogenous regulator and is absolutely necessary for FN expression in primary human skin dermal fibroblasts. CCN2 is significantly induced by Smad3, and functions an important regulator of FN gene expression. These findings provide new insight into information that CCN2 is responsible for mediating the stimulatory effects of TGF-β/Smad3 on FN gene expression.

## Materials and methods

### Materials

Dulbecco’s Modified Eagle’s Media (DMEM), fetal bovine serum, trypsin solution, penicillin, and streptomycin were purchased from Invitrogen Life Technology (Carlsbad, CA). Antibodies for CCN2 (SC-14939), FN (SC-18827), Smad2/Smad3 (SC-8332) were purchased from Santa Cruz Biotechnology (Santa Cruz, CA). β-actin antibody was purchased from Sigma Chemical (St. Louis, MO). Human recombinant TGF-β1 was purchased from R&D Systems (Minneapolis, MN). Unless otherwise stated, all other reagents were purchased from Sigma Chemical Company (St. Louis, MO).

### Ethics statement

This study was conducted in compliance with Declaration of Helsinki principles. All procedures involving human subjects were approved by the University of Michigan Institutional Review Board, and all subjects provided written informed consent.

### Procurement of human tissue samples

Normal skin punch biopsies (4 mm) were obtained from sun-protected buttock skin (aged 41–52 years), as previously described [18]. Completely de-identified SCC samples were obtained from the University of Michigan Cutaneous Oncology Unit. For CO_2_ laser ablation, wounds were created on focal areas of the forearm skin using a carbon dioxide (CO_2_) laser (Ultrapulse; Coherent, Santa-Clara, CA) [19]. Full-thickness punch biopsies (4 mm) were performed in a non-treated area as a control and in the center of wounded areas at various time points after treatment. All freshly obtained biopsies were embedded in OCT, frozen in liquid nitrogen, and stored at -80°C until processing.

### Cell culture

Human primary dermal fibroblasts were established by outgrowth from human skin punch biopsies of healthy adult volunteers (aged 21–52 years), as described previously [20]. Cells were cultivated in DMEM supplemented with 10% Fetal Bovine Serum (Invitrogen, Carlsbad, CA). Cells were plated at 70–80% confluence, and used one day later. Cells were utilized between passages 4 and 10. Independent replicates of studies, indicated by N number in figure legends, were performed with cells from different individuals.

### Laser capture microdissection, RNA isolation, and quantitative real-time RT-PCR

Human epidermis and dermis were captured by laser capture microdissection (LCM) (Leica ASLMD system; Leica Microsystems, Wetzlar, Germany). Briefly, skin samples embedded in OCT and sectioned (15 μm), stained with hematoxylin and eosin. Total RNA was prepared from LCM-captured tissues using a commercial kit (RNeasy Micro kit, Qiagen, Chatsworth, CA). The quality and quantity of total RNA were determined by Agilent 2100 bioanalyzer (Agilent Technologies, Santa Clara, CA). Total RNA from cells was extracted using TRIzol reagent (Invitrogen, Carlsbad, CA). cDNA for PCR templates was prepared by reverse transcription of total RNA (100 ng) using Taqman Reverse Transcription kit (Applied Biosystems, Carlsbad, CA). Real-time PCR was performed on a 7300 Sequence Detector (Applied Biosystems, Carlsbad, CA) using Taqman Universal PCR Master Mix Reagents (Applied Biosystems, Carlsbad, CA). All real-time PCR primers were designed and purchased from Sigma and the sequences were reported in our previous publications [6]. Target gene mRNA expression levels were normalized to the housekeeping gene 36B4 (acidic ribosomal phosphoprotein P0) [21] as an internal control for quantification.

### Immunohistology

Immunohistology was performed as described previously [22]. Briefly, skin samples embedded in OCT were sectioned (7μm), fixed in 2% paraformaldehyde, permeabilized with 0.5% Triton X-100 in phosphate-buffered saline (PBS), blocked with rabbit serum (5% in PBS), and incubated for one hour at room temperature with CCN2 and FN antibodies (Santa Cruz Biotechnology, Santa Cruz, CA), followed by incubation with secondary antibody for one hour at room temperature. After staining, the slides were examined using a digital imaging microscope (Zeiss, Germany). Specificity of staining was determined by substituting isotype-control immunoglobulin (mouse IgG2a) for the primary antibodies. No detectable staining was observed with isotype-controls (data not shown).

### Western analysis

Proteins were extracted from cells by whole cell extraction buffer (25mM HEPES [pH 7.7], 0.3 M NaCl, 1.5 mM MgCl2, 0.2 mM EDTA, 0.1% Triton X-100, 0.5 mM DTT, 20 mM β-glycerolphosphate, 0.1 mM Na3VO4, 2μg/ml leupeptin, and 100μg/ml PMSF) and equal amounts of protein (~50μg/lane) were analyzed by resolving on 10–12% sodium dodecyl sulfate-polyacrylamide (SDS) gel electrophoresis. The SDS gels were transferred to polyvinylidene difluoride membrane, and the membranes were blocked with PBST (0.1% Tween 20 in PBS) containing 5% nonfat milk for one hour at room temperature. Primary antibodies (Santa Cruz Biotechnology, Santa Cruz CA) were incubated with the polyvinylidene difluoride membrane for one to two hours at room temperature, after which membranes were washed three times with PBST solution and incubated with appropriate secondary antibodies for one hour at room temperature. After washing three times with PBST, the membranes were developed with ECF (Vistra ECF Western blotting system, GE Health Care, Piscataway, NJ) following the manufacturer’s protocol. The membranes were scanned with a STORM MolecularImager (Molecular Dynamics, Sunnyvale, CA), the fluorescence intensities of each band were quantified by ImageQuant (GE Health Care, Piscataway, NJ) and normalized using β-actin as a marker for equal protein loading.

### ProteinSimple capillary electrophoresis immunoassay

Whole cells extract was prepared as described above in Western analysis. ProteinSimple capillary electrophoresis immunoassay was performed according to the ProteinSimple user manual. In brief, whole cell extract samples (800 ng/lane) were mixed with a master mix (ProteinSimple) to a final concentration of 1x sample buffer, 1x fluorescent molecular weight markers, and 40 mM dithiothreitol (DTT) and then heated at 95°C for 5 min. The samples, blocking reagent, primary antibodies, HRP-conjugated secondary antibodies, chemiluminescent substrate, and separation and stacking matrices were also dispensed to designated wells plate. The electrophoresis and immunodetection steps took place in the capillary system (ProteinSimple Wes, ProteinSimple, Santa Clare, CA, USA) and were fully automated with instrument default settings. The digital image was analyzed and quantified with Compass software (ProteinSimple, Santa Clare, CA, USA) after normalization by and β-actin (loading control).

### Transfection

Human primary skin fibroblasts were transiently transfected by electroporation (Amaxa, Koeln, Germany). Transient transfection of Emerald Green Fluorescent Protein (EmGFP, The Vivid Colors^™^ ThermoFisher ‎Waltham, MA) indicated that the transfection efficiency can be up to 80% in human primary skin fibroblasts by electroporation. After transfection (48 hours), total RNA and cellular protein were extracted and mRNA and protein levels were determined by real-time RT-PCR and Western analysis, respectively, as described above. #1 siRNAs for CCN2 (AAG-TAC-CAG-TGC-ACG-TGC-CTG), Smad2 (AAT-TTG-GGG-ACT-GAG-TAC-ACC), and Smad3 (AAA-CCT-ATC-CCC-GAA-TCC-GAT) were designed from human mRNA open reading frames according to OligoEngine (Seattle, WA) website instructions and submitted to a BLAST search against human genome database to confirm specificity. #1siRNA for CCN2, Smad2, and Smad3 were synthesized by Qiagen (Chatsworth, CA). #2 and #3 si RNAs for CCN2, Smad2, and Smad3 were purchased from Dharmacon (Lafayette, CO). Control siRNA (AAT-TGT-CCG-AAC-GTG-TCA-CGT) were purchased from Qiagen (Chatsworth, CA). Smad2 and Smad3 expression vectors were kindly provided by Dr. Massague (Memorial Sloan-Kettering Cancer Center, New York). The CCN2 siRNA target specificity was confirmed further using “rescue” experiments, in which the CCN2 protein was re-expressed from a transiently transfected CCN2 vector that encodes an altered mRNA that is resistant to the siRNA silencing. CCN2 siRNA resistance expression vector was generated by introducing two point mutations from CCN2 siRNA sequence (AAG-TAC-GTG-TGC-ACG-TGC-CTG, underlined nucleotides indicate introduced point mutation from CA to GT) from CCN2 expression vector (CCN2-V5 TOPO). CCN2 expression vector (CCN2-V5 TOPO) [23] was generously provided by Dr. Wahab (Cell and Molecular Biology Section, Imperial College School of Medicine, London). In all experiments, 1 × 10^6^ cells were transfected with siRNAs (20 nM) by electroporation (Amaxa, Koeln, Germany). For plasmid transfection, 1 × 10^6^ cells were transfected with 1μg of expression vectors and control vector (pCDNA3.1, Invitrogen, Carlsbad, CA) by electroporation (Amaxa, Koeln, Germany). In some experiments, varying amounts of expression vectors were transfected, as indicated in the figure legends.

### Statistical analysis

Comparisons were made with the paired *t*-test (two groups) or the repeated measures of ANOVA (more than two groups). Multiple pair-wise comparisons were made with the Tukey Studentized Range test. All p values are two-tailed, and considered significant when <0.05.

## Result

### FN expression is dependent on endogenous CCN2 in primary dermal fibroblasts

**Fig 1:**
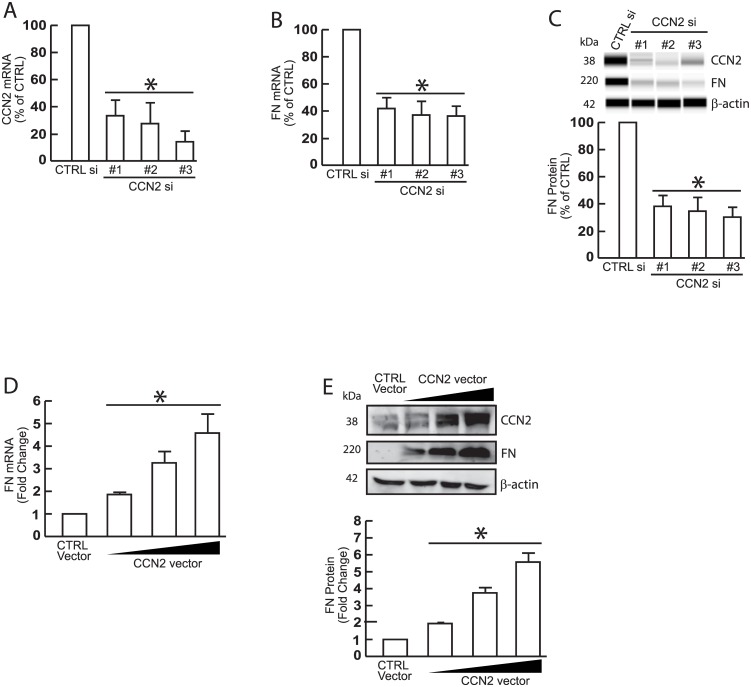
FN expression is regulated by CCN2 in primary dermal fibroblasts. Primary dermal fibroblasts (1 × 10^6^) were transfected with non-specific control siRNA or CCN2 siRNAs (20nM) (A, B, C), or control vector (pCDNA3.1, 2μg) or increasing amounts of CCN2 vector (0.5, 1, and 2μg) (D, E). Total RNA and whole cell extract were prepared 48 hours after transfection. (A) CCN2 mRNA levels. (B) FN mRNA levels. (C) CCN and FN Protein levels. (D) FN mRNA levels. (E) CCN and FN protein levels. mRNA levels were quantified by real-time RT-PCR. Protein levels were determined by ProteinSimple capillary electrophoresis immunoassay (C) and Western analysis (E). mRNA levels were normalized to mRNA for 36B4, a ribosomal protein used as an internal control quantitation. Protein levels were normalized by β-actin (loading control). Insets show representative digital images (C) and Western blots (E). Data are expressed as mean±SEM, N = 3–5, *p<0.05.

In order to directly address the role of CCN2 in regulating FN, we employed gain- and loss-of-function approaches of CCN2 in primary dermal fibroblasts, the major cells are responsible ECM production in skin. siRNA-mediated knockdown of endogenous CCN2 (Fig 1A) significantly reduced the levels of FN mRNA (Fig 1B) and protein (Fig 1C). In contrast, overexpression of CCN2 increased the expression levels of FN in mRNA (Fig 1D) and protein (Fig 1E) in a dose dependent manner, indicating that CCN2 is an endogenous stimulator for FN gene expression in primary dermal fibroblasts.

### CCN2 acts as a mediator of TGF-β induced FN in primary dermal fibroblasts

**Fig 2:**
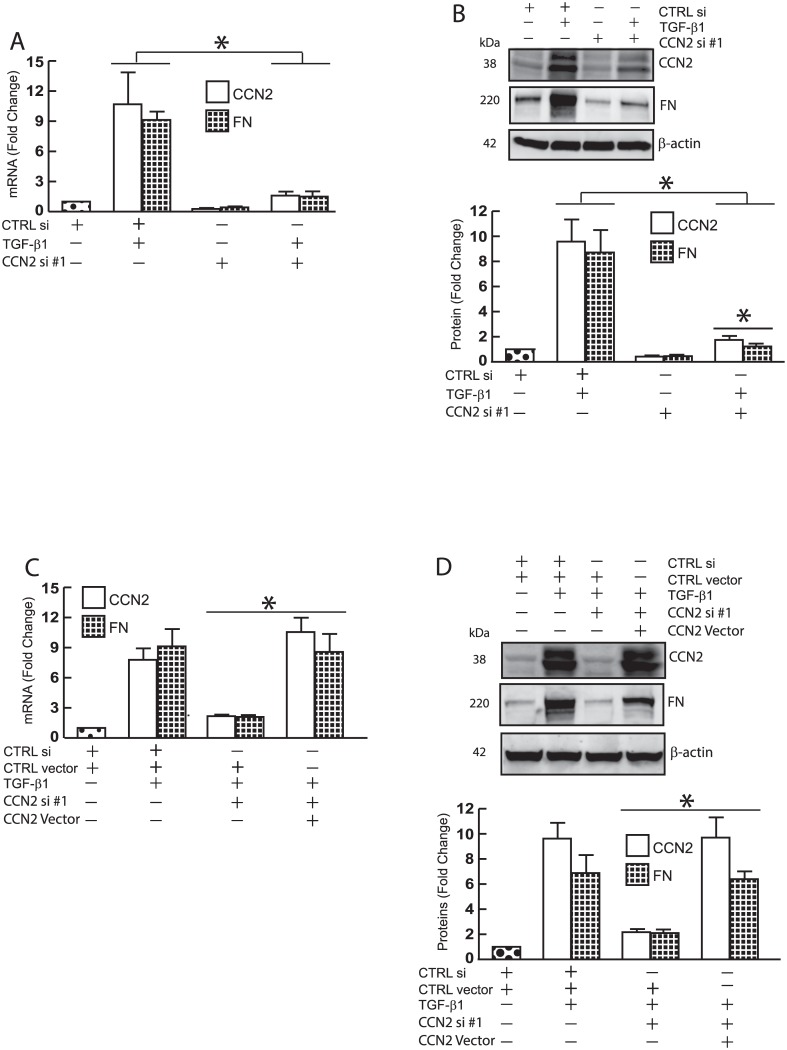
TGF-β-dependent FN expression is mediated by CCN2 in primary dermal fibroblasts. Primary dermal fibroblasts (1 × 10^6^) were transfected with the indicated siRNAs (20nM) and plasmid vectors (1μg). 32 hours after transfection, cell were treated with TGF-β1 (5 ng/ml) for 16 hours. Total RNA and whole cell extract were prepared 48 hours after transfection. mRNA and protein levels were quantified by real-time RT-PCR and Western analysis, respectively. mRNA levels were normalized to mRNA for 36B4, a ribosomal protein used as an internal control for quantitation. Protein levels were normalized by β-actin (loading control). Insets show representative Western blots. (A, C) CCN2 and FN mRNA. (B, D) CCN2 and FN proteins. Data are expressed as mean±SEM, N = 3. *p<0.05 vs control.

CCN2 is well-known as a down-stream mediator of TGF-β signaling [6], which is a primary regulator of FN expression [24]. Thus, we explored the role of CCN2 in TGF-β induced FN expression. As both CCN2 and FN are markedly induced by TGF-β1, knockdown of CCN2 by CCN2 siRNA resulted in significant inhibition of FN mRNA (Fig 2A) and protein (Fig 2B). Importantly, FN inhibition by CCN2 was rescued by co-transfection of a plasmid containing an expression cassette for CCN2 harboring silent mutations rendering it invulnerable to the siRNA treatment (Fig 2C and 2D, last lanes, see Materials and methods for details of CCN2 siRNA resistance expression vector). These data demonstrate that CCN2 functions as a mediator of TGF-β induced FN expression in primary dermal fibroblast.

### CCN2 and FN are regulated by Smad3 in primary dermal fibroblasts

**Fig 3:**
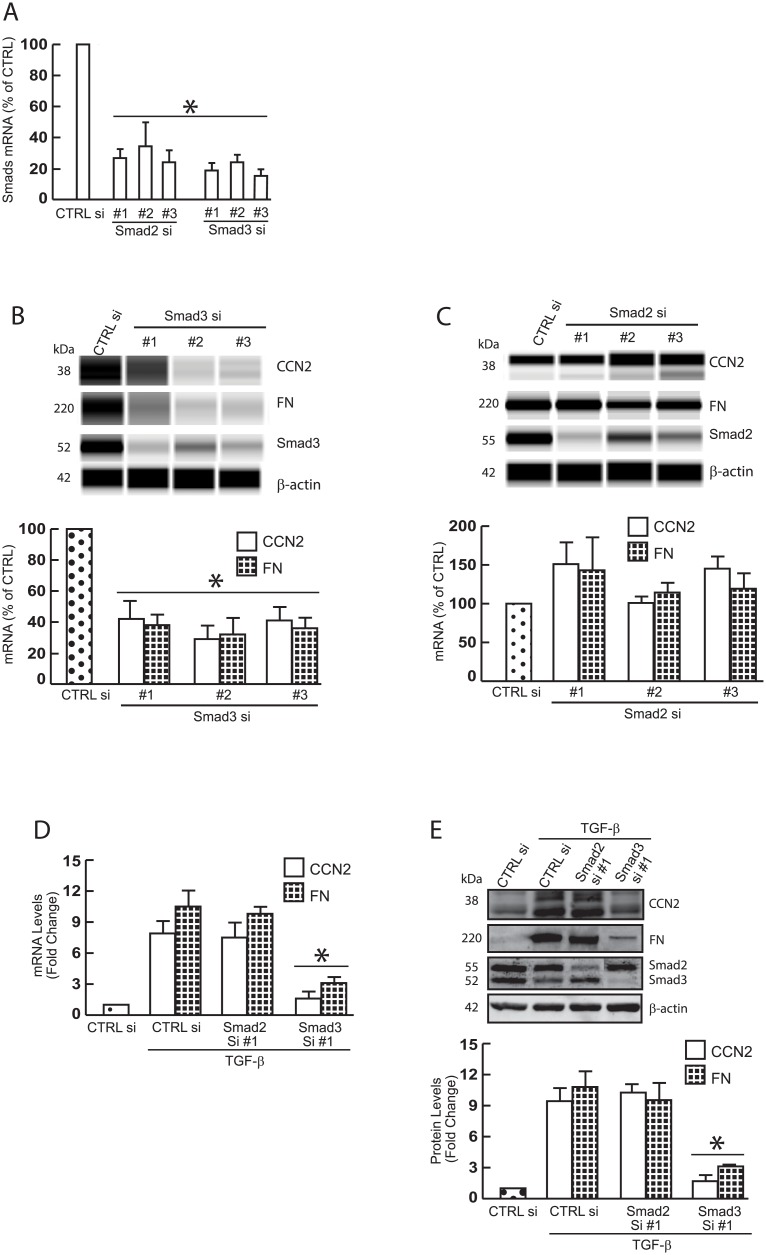
CCN2 and FN expression are primarily regulated by Smad3 in primary dermal fibroblasts. Primary dermal fibroblasts (1 × 10^6^) were transfected with the indicated siRNAs (20nM). Total RNA and whole cell extract were prepared 48 hours after transfection. mRNA levels were quantified by real-time RT-PCR. Protein levels were determined by ProteinSimple capillary electrophoresis immunoassay (B, C) and Western blots (E). mRNA levels were normalized to mRNA for 36B4, a ribosomal protein used as an internal control for quantitation. Protein levels were normalized by β-actin (loading control). Insets show representative digital images (B, C) and Western blots (E). (A) Smad2 and Smad3 mRNA levels. (B) CCN2, FN, and Smad3 protein levels. (C) CCN2, FN, and Smad2 protein levels. (D) CCN2 and FN mRNA levels. 32 hours after transfection, cell were treated with TGF-β1 (5 ng/ml) for 16 hours. (E) CCN2, FN, Smad2, and Smad3 protein levels. 32 hours after transfection, cell were treated with TGF-β1 (5 ng/ml) for 16 hours. Data are expressed as mean±SEM, N = 3, *p<0.05 vs control.

Two TGF-β receptor-activated Smad transcription factors, Smad2 and Smad3, function as important intracellular signal transducers of TGF-β signaling pathway [25]. However, it is not known which Smad proteins regulate CCN2 and FN in primary dermal fibroblasts. Thus, we undertook to determine the specific Smad proteins that regulate the expression of CCN2 and FN. To achieve this goal, endogenous Smad2 and Smad3 were knocked-down by Smad2 and Smad3 specific siRNAs, respectively (Fig 3A). siRNA-mediated knockdown of Smad3 (Fig 3B), but not Smad2 (Fig 3C), reduced protein levels of CCN2 and FN. In agreement with these data, TGF-β1-stimulated CCN2 and FN mRNA (Fig 3E) and protein (Fig 3F) were significantly abolished by knockdown of endogenous Smad3, but not Smad2. These data demonstrate that Smad3 is required for CCN2 and FN expression in primary dermal fibroblasts.

### Smad3-dependent regulation of FN is mediated by CCN2 in primary dermal fibroblasts

**Fig 4:**
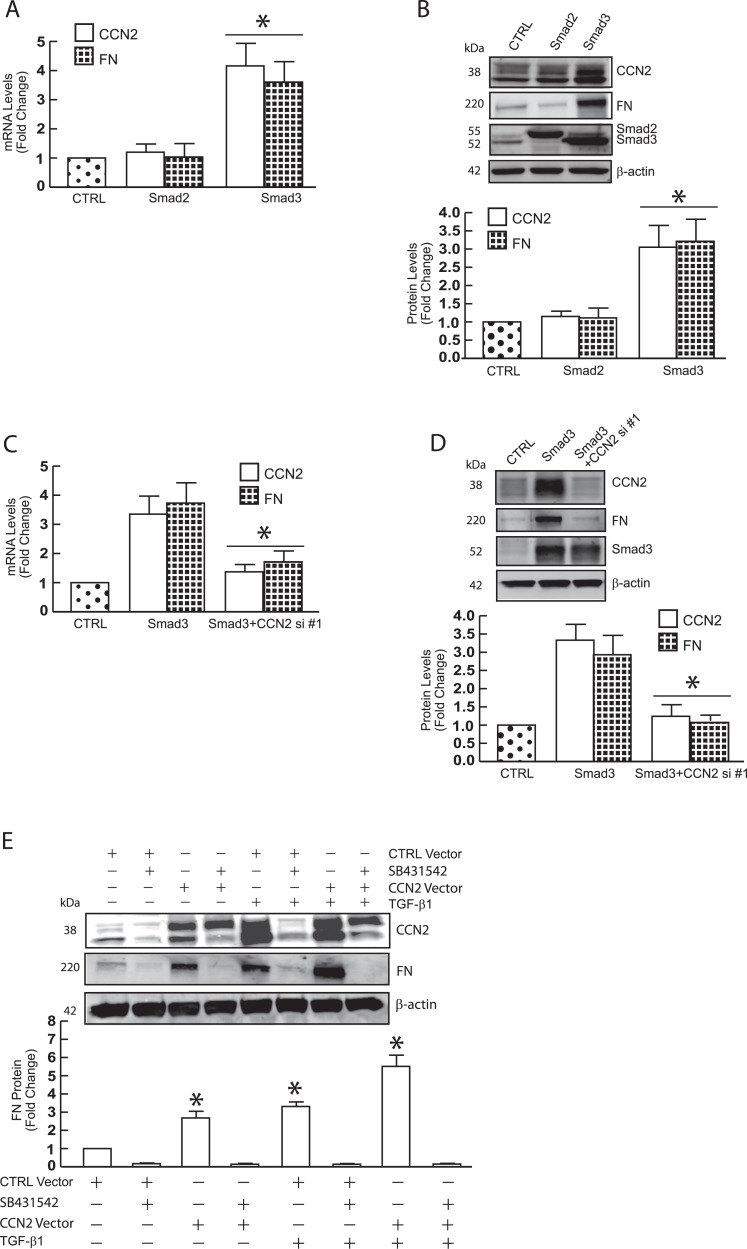
CCN2 functions primary mediator in Smad3-dependent expression of FN in primary dermal fibroblasts. Primary dermal fibroblasts (1 × 10^6^) were transfected with the indicated siRNAs (20nM) and vectors (1μg). Total RNA and whole cell extract were prepared 48 hours after transfection. mRNA and protein levels were quantified by real-time RT-PCR and Western analysis, respectively. mRNA levels were normalized to mRNA for 36B4, a ribosomal protein used as an internal control for quantitation. Protein levels were normalized by β-actin (loading control). Insets show representative Western blots. (A, C) CCN2 and FN mRNA levels. (B, D) CCN2, FN, Smad2, and Smad3 protein levels. (E) The ability of CCN2 to regulate FN expression is dependent on intact TGF-β signaling. 32 hours after transfection, cell were treated with TGF-β1 (5 ng/ml) for 16 hours. Data are expressed as mean±SEM, N = 3, *p<0.05 vs control.

In contrast to knockdown approaches, over-expressing Smad3, but not Smad2, increased expression of CCN2 and FN mRNA (Fig 4A) and protein (Fig 4B). Furthermore, knockdown of CCN2 resulted in significant blockage of Smad3-induced FN in both mRNA (Fig 4C) and protein levels (Fig 4B). These data demonstrate that Smad3 and CCN2 act in concert to regulate FN production in primary dermal fibroblasts. Next, we explored the potential mechanism in which CCN2 stimulates FN expression. As described (Fig 1C and 1D), overexpression of CCN2 resulted in significant stimulation of FN expression and that was further increased by TGF-β1 treatment (Fig 4E, third and seventh lanes). Interestingly, CCN2 is unable to stimulate FN expression when TGF-β signaling is blocked by a specific inhibitor of TGF-β pathway (TGF-β type I receptor, SB431542) (Fig 4F, fourth and last lanes). These data indicate that the ability of CCN2 to stimulate FN expression is dependent on intact TGF-β signaling in human dermal fibroblasts.

### Predominant expression of CCN2 and FN in the dermis of normal human skin, stromal tissues of skin Squamous-Cell Carcinoma (SCC), and wounded human skin

**Fig 5:**
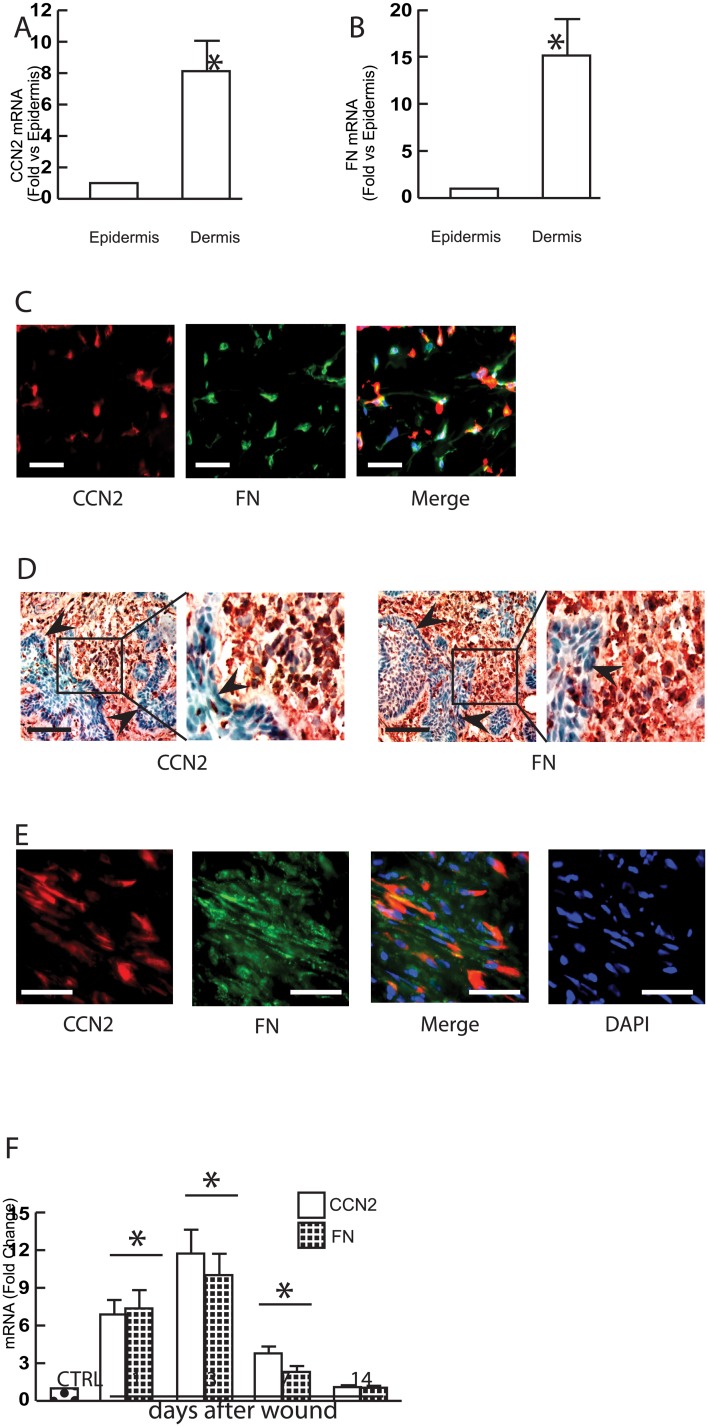
CCN2 and FN are primarily expressed in the dermis of normal human skin, stromal tissues of skin SCC, and wounded human skin. (A, B) Epidermis and dermis were captured by LCM. Total RNA was extracted from captured tissue, and mRNA levels were quantified by real-time RT-PCR. CCN2 (A) and FN (B) mRNA levels were normalized to the housekeeping gene 36B4, as an internal control for quantification. Data are relative levels to 36B4 (mean±SEM), N = 6, *p<0.05. (C) Double immunostaining for CCN2 and FN in normal human skin. OCT-embedded normal human skin sections (7μm) were co-immunofluorescence stained with CCN2 and FN. Representative of six individuals. Bar = 50μm. (D) Expression of CCN2 and FN in the stromal tissues of SCC was determined by immunohistology. Arrow heads indicate tumor islands. Representative of five SCC. Bar = 100μm. (E) Double immunostaining for CCN2 and FN. Representative of six individuals. Bar = 50μm. (F) Partial thickness wounds were made in forearm skin of healthy adult individuals by CO_2_ laser (see Methods for details). Skin samples were obtained at indicated times, and mRNA levels were quantified by real-time RT-PCR. CCN2 and FN mRNA levels were normalized to the housekeeping gene 36B4, as an internal control for quantification. Mean±SEM, N = 6, *p<0.05 vs control.

Finally, we determined the CCN2 and FN expression in normal human skin, SCC human tissues, and wounded human skin. Laser capture microdissection indicated that CCN2 (Fig 5A) and FN (Fig 5B) were predominantly expressed in the dermis of normal human skin. Double immunostaining further indicated that CCN2 partially co-localized with FN, a marker of dermal fibroblasts (Fig 5C). In SCC skin tissues, CCN2 (Fig 5D, left panel) and FN (Fig 5D, right panels) are markedly overexpressed in the stromal tissue of SCC. Double immunostaining for CCN2 and FN revealed partial co-localization (Fig 5E). We also found that CO_2_ laser ablation, which causes partial thickness wound, rapidly and markedly induces both CCN2 and FN expression in human skin dermis *in vivo* (Fig 5F). Importantly, the timing of elevated CCN2 levels nicely corresponds to the FN induction by wound healing response in human skin *in vivo*. These data demonstrate that CCN2 and FN are predominantly expressed in human skin dermal stromal tissues, and support the possibility that CCN2 stimulates FN expression in normal human skin, wounded human skin, and the stromal tissue of SCC.

## Discussion

In the present study, we investigated the role of CCN2 in FN expression in human skin dermal fibroblasts, the major cells responsible for ECM homeostasis. We provide evidence that CCN2 functions as an intrinsic, endogenous physiological mediator of FN expression.

FN is an important ECM constituent and plays a major role in cell adhesion, growth, migration, and differentiation [26, 27]. FN plays a crucial role wound healing [28, 29] and is necessary for embryogenesis [30]. Altered FN expression has been associated with a number of pathologies, including cancer [31] and fibrosis [32]. In skin, dermal fibroblasts are major source of FN production. In skin, FN plays an important role in many critical biologic activities, including cell-to-cell adhesion, cell-to-ECM adhesion, migration and differentiation, maintenance of cellular structure and shape, wound healing, and blood coagulation. It is also well known that FN is important in maintenance of ECM homeostasis by interactions with collagen, heparan sulfate, fibrin, and hyaluronic acid [33]. Although the TGF-β pathway is a well-known primary regulator of FN expression, less is known of its down-stream mediators, such as Smads and CCN2 proteins, in mediating FN stimulation in response to TGF-β signaling. Our data reveal that CCN2 expression is largely regulated by Smad3, and acts as an important mediator of FN gene expression in normal human skin dermis and in fibrotic ECM micronenvironment in skin SCC.

The mechanism by which CCN2 stimulates FN expression in human skin dermal fibroblasts is not known. Emerging evidence indicates that the actions of CCN2 are mediated through its interactions with multiple integrins including α5β1 [34], αvβ3 [35], α6β1 [36]; [37], α4β1 [38], αMβ2 [39] and αIIbβ3 [40] in a cell type specific manner. This binding can activate integrin signaling pathways leading to activation of focal adhesion kinase and MAP kinases. CCN2 has also been reported to bind to ECM heparan sulfate proteoglycans perlecan [41] and syndecan 4 [38]. These data support the concept that CCN2 cooperates with ECM proteins to specify functional interactions with their cell surface receptors [42]. Additional studies are warranted to uncover the precise molecular mechanism(s) by which CCN2 regulates FN expression.

It has been reported that CCN2 enhances the binding of TGF-β ligands to its receptor complex, thereby stimulating intracellular Smad2/3 phosphorylation and Smad-dependent transcriptional activity, in mink lung epithelial cells [43]. However, we have previously reported that in human skin dermal fibroblasts, either CCN2 knockdown or over-expression had no effect on Smad2/Smad3 phosphorylation and TGF-β/Smad3-dependent reporter gene [6]. These data indicate that the ability of endogenous CCN2 to regulate FN expression is not dependent on direct potentiation of Smad activation in human dermal fibroblasts. Shi-Wen et al have reported that CCN2 action is Smad independent [15]. Interestingly, we found that the ability of CCN2 to upregulate FN expression is dependent on intact TGF-β signaling (Fig 4F). Recently Qi et al reported that the profibrotic effect of CCN2 was completely abrogated in the presence of pan-specific TGF-β and TGF-β type II receptor neutralizing antibodies in renal cortical fibroblasts [44], indicating that CCN2 requires intact TGF-β signaling to exert its effect on ECM production. CCN2 is potently induced by TGF-β and this increased CCN2 stimulates FN expression. Thus, CCN2 is both regulated by TGF-β signaling, and is required for downstream action of TGF-β.

It is well documented that CCN2 is rapidly and potently induced by TGF-β in a variety of cells including primary human skin fibroblasts [5, 45, 46]. We found that CCN2 acts as a downstream mediator of TGF-β/Smad signaling and acting synergistically with TGFβ to regulate FN gene expression. Synergy between CCN2 and TGF-β has been reported in a mouse fibrosis model [47]. Injection of TGF-β or CCN2 alone into mouse skin caused transient fibrotic tissue formation. However, simultaneous injection of TGF-β plus CCN2 produced long-term, persistent fibrotic tissue formation. Consistent with these data, recently Chujo *et al* reported that serial subcutaneous injection of CCN2 after TGF-β resulted in a significant increase of COL1A2 promoter activity and mRNA expression, compared with TGF-β treatment alone [48]. CCN2 has also been shown to induce COL1A2 promoter activity in proximal tubular epithelial cells [49]. Furthermore, Yang *et al* [50] demonstrated that CCN2 augments TGF-β-induced myofibroblast differentiation in normal rat kidney fibroblasts *in vitro*. In human skin wound, the timing of elevated CCN2 levels nicely corresponds to the FN induction. These data suggest that CCN2 may function as a stimulatory factor in FN gene expression in response to wound in human skin. However, it appears that CCN2 itself is not required for skin wound healing in mouse model, since loss of CCN2 does not affect wound closure kinetics [51].

As Smad proteins play pivotal role in TGF-β-dependent regulation of ECM production, little is known about the role of specific Smad protein in CCN2 and FN gene expression. Our data demonstrate that Smad3 functions a key molecule in mediating TGF-β stimulation of CCN2 and FN in human skin fibroblasts. Either knockdown or overexpression of Smad2 does not alter CCN2 and FN expression (Figs 3 and 4). The expression of Smad2 in primary human skin dermal fibroblasts is relatively high (Fig 3B, 3D and 3F), and we achieved incomplete knockdown of Smad2 (76%, Fig 3A). These data cannot rule out the possibility that a Smad2/3 heterodimer may be needed for FN expression. CCN2 is much lower in Smad3 knockout mice, and importantly TGF-β was unable to induce CCN2 in Smad3 null mice [52]. These findings are consistent with the notion that stimulation of CCN2 by TGF-β is Smad3-dependent. It has been suggested that Smad2 and Smad3 exhibit distinct roles, and function differently, in TGF-β-dependent gene expression [53–55]. In general, Smad3 is the essential mediator of TGF-β immediate-early target genes, such as transcription factors, for example JunB, c-fos c-Myc. Inhibition of Smad3 but not Smad2 allows keratinocytes to escape from TGF-β induced cell cycle arrest. In contrast, in some genes such as matrix metalloproteinases, MMP-2 expression is Smad2 dependent but not Smad3. On the other hand, some genes such as plasminogen activator inhibitor-1 (PIA-1) require both Smad2 and Smad3. One of the significant physical differences between Smad2 and Smad3 is their DNA binding activity. Smad3 is able to bind directly to the TGF-β target gene promoter but not Smad2 [25]. The data from Smad2 and Smad3 knockout mice further suggested that individual Smad plays different roles during embryonic development [56]. Deletion of Smad2 is embryonic lethal due to failure to establish an anterior-posterior axis, gastrulation, and mesoderm formation, whereas Smad3 knockout mice are viable and survive several months, suggesting that Smad3 is dispensable for embryonic development. In supporting our data, targeted disruption of Smad3 significantly attenuates pulmonary, renal, and skin fibrosis in a mouse models, suggesting that Smad3 plays an important role in collagen homeostasis [52, 57–60].

Fetal bovine serum (FBS)-supplemented medium is routinely used for cell culture including primary human skin dermal fibroblasts. However, FBS often contains a high level of TGF-β and other poorly defined factors that may hamper the correct measurement of endpoint. Because of this, some investigators often use serum-free medium in order to avoid contribution of FBS-derived undefined factors in the culture medium. However, we found that under serum starvation or serum reduced (0.5% FBS) conditions, transfected primary human skin dermal fibroblasts, unlike immortalized cell lines, undergo apoptosis and do not respond well to TGF-β1 and other stimulators. Commercially available common serum-free or serum-reduced media, such as Medium 106 or OPTI-MEM (ThermoFisher/Invitrogen), often do not support primary human skin dermal fibroblasts growth *in vitro*. Due to these reasons, all cell experiments that are involved in the current manuscript were performed under serum (10%) containing tissue culture condition. Therefore, our data do not rule out the possibility that CCN2-dependent events could be due to synergy with TGF-β and some undefined serum components.

In summary, CCN2 is necessary for FN expression in primary human skin dermal fibroblasts. CCN2 acts as a downstream mediator TGF-β/Smad signaling to regulate FN gene expression in normal human skin dermis, wounded human skin, and in fibrotic ECM microenvironment in SCC. These findings provide new insight into information that CCN2 functions an important mediator in mediating the stimulatory effects of TGF-β/Smad3 on FN expression in human skin.
