# Correction: Smad3-dependent CCN2 mediates fibronectin expression in human skin dermal fibroblasts

**DOI:** 10.1371/journal.pone.0177611

**Published:** 2017-05-09

**Authors:** The PLOS ONE Staff

Elements of Fig 5A, 5B and 5F are incorrectly offset. Please see the corrected Fig 5 here. The publisher apologizes for the error.

**Fig 5:**
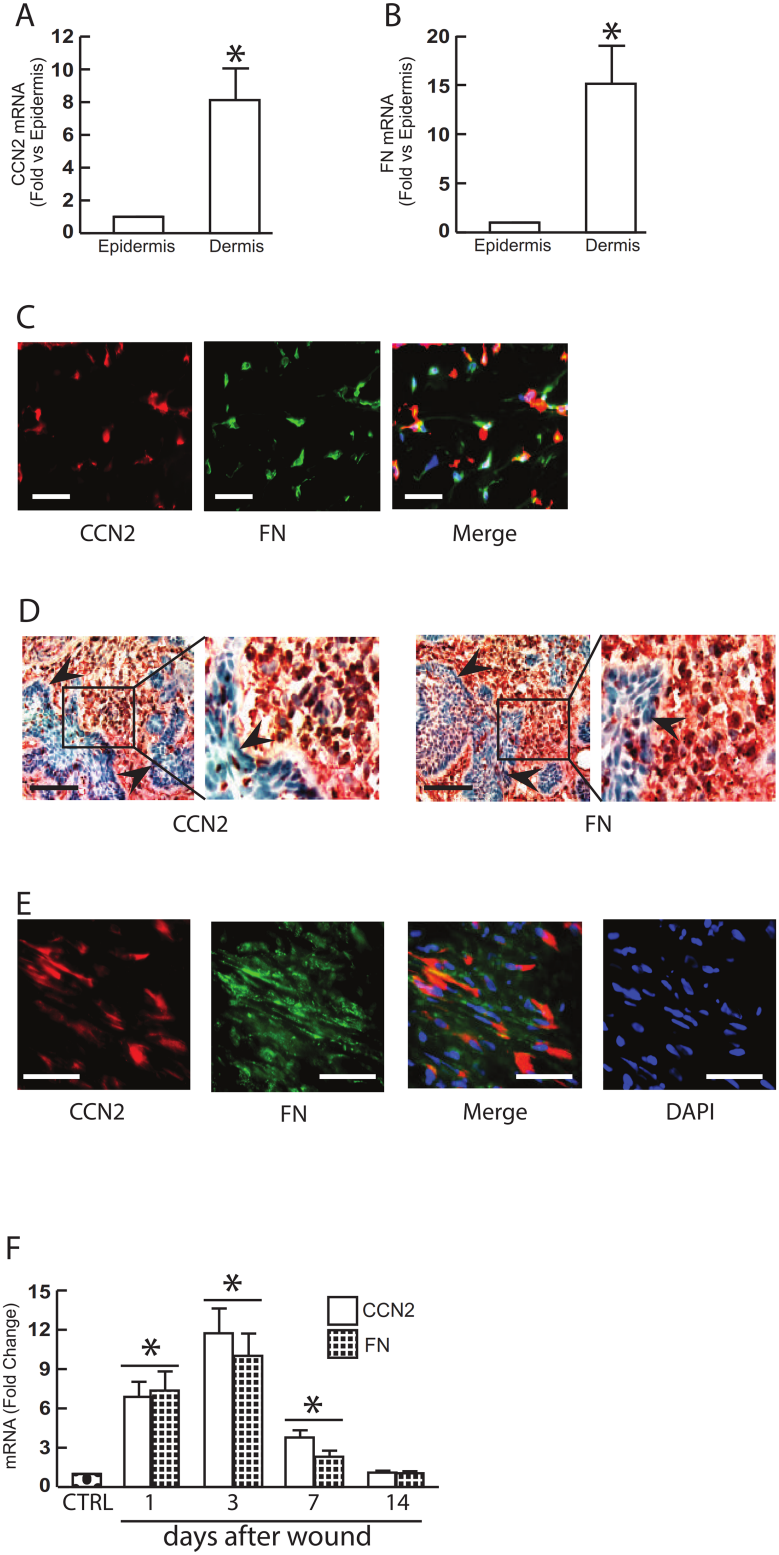
CCN2 and FN are primarily expressed in the dermis of normal human skin, stromal tissues of skin SCC, and wounded human skin. (A, B) Epidermis and dermis were captured by LCM. Total RNA was extracted from captured tissue, and mRNA levels were quantified by real-time RT-PCR. CCN2 (A) and FN (B) mRNA levels were normalized to the housekeeping gene 36B4, as an internal control for quantification. Data are relative levels to 36B4 (mean±SEM), N = 6, *p<0.05. (C) Double immunostaining for CCN2 and FN in normal human skin. OCT-embedded normal human skin sections (7μm) were co-immunofluorescence stained with CCN2 and FN. Representative of six individuals. Bar = 50μm. (D) Expression of CCN2 and FN in the stromal tissues of SCC was determined by immunohistology. Arrow heads indicate tumor islands. Representative of five SCC. Bar = 100μm. (E) Double immunostaining for CCN2 and FN. Representative of six individuals. Bar = 50μm. (F) Partial thickness wounds were made in forearm skin of healthy adult individuals by CO_2_ laser (see Methods for details). Skin samples were obtained at indicated times, and mRNA levels were quantified by real-time RT-PCR. CCN2 and FN mRNA levels were normalized to the housekeeping gene 36B4, as an internal control for quantification. Mean±SEM, N = 6, *p<0.05 vs control.
